# Developing and validating risk prediction models in an individual participant data meta-analysis

**DOI:** 10.1186/1471-2288-14-3

**Published:** 2014-01-08

**Authors:** Ikhlaaq Ahmed, Thomas PA Debray, Karel GM Moons, Richard D Riley

**Affiliations:** 1 Midlands Hub for Trials Methodology Research, School of Health and Population Sciences, University of Birmingham, Edgbaston, Birmingham B15 2TT, UK; 2 Julius Center for Health Sciences and Primary Care, University Medical Center Utrecht, Utrecht, The Netherlands; 3 School of Health and Population Sciences, Public Health Building, University of Birmingham, Edgbaston, Birmingham B15 2TT, UK

**Keywords:** Meta-analysis, Prognostic factor, Prognosis, Individual participant (patient) data, Review, Reporting

## Abstract

**Background:**

Risk prediction models estimate the risk of developing future outcomes for individuals based on one or more underlying characteristics (predictors). We review how researchers develop and validate risk prediction models within an individual participant data (IPD) meta-analysis, in order to assess the feasibility and conduct of the approach.

**Methods:**

A qualitative review of the aims, methodology, and reporting in 15 articles that developed a risk prediction model using IPD from multiple studies.

**Results:**

The IPD approach offers many opportunities but methodological challenges exist, including: unavailability of requested IPD, missing patient data and predictors, and between-study heterogeneity in methods of measurement, outcome definitions and predictor effects. Most articles develop their model using IPD from *all* available studies and perform only an internal validation (on the same set of data). Ten of the 15 articles did not allow for any study differences in baseline risk (intercepts), potentially limiting their model’s applicability and performance in some populations. Only two articles used external validation (on different data), including a novel method which develops the model on all but one of the IPD studies, tests performance in the excluded study, and repeats by rotating the omitted study.

**Conclusions:**

An IPD meta-analysis offers unique opportunities for risk prediction research. Researchers can make more of this by allowing separate model intercept terms for each study (population) to improve generalisability, and by using ‘internal-external cross-validation’ to simultaneously develop and validate their model. Methodological challenges can be reduced by prospectively planned collaborations that share IPD for risk prediction.

## Background

One of the cornerstones of health and clinical research is to identify individuals who have a high risk of developing an adverse outcome over a specific time period, so that they can be targeted for early preventative strategies and possibly treatment. For example individuals who are seemingly healthy but are found to have a high risk of developing cardiovascular disease could be recommended to modify their lifestyle and behaviour (e.g. smoking, exercise, eating habits) to reduce their future risk. They may also be prioritised for clinical investigation, which could lead to early diagnosis of an underlying condition (e.g. diabetes, high blood pressure) and preventative treatment (e.g. statins or aspirin) to manage it.

For this purpose of prognostic risk assessments there is a growing interest in *risk prediction modelling, *[1-3] where a statistical model is used to estimate the risk of future outcomes for individuals based on one or more underlying characteristics. When considering future outcomes in patients, a risk prediction model is often referred to as a *prognostic model* (typically used for outcome risk for a defined disease) or more generally a *clinical prediction model* (used for both diseased or non-diseased settings) Similarly the word ‘model’ is often replaced with ‘score’, ‘tool’, ‘index’, or ‘rule’. However, the same principle remains: to accurately predict the risk of future occurrence of an outcome in an individual by utilising the values or levels of multiple individual characteristics. We refer here to such characteristics simply as predictors, but they are also termed prognostic factors, risk factors, prognostic variables, and prognostic markers [4]. They often include standard features such as age, sex, smoking and family history, but also increasingly include more complex clinical measures such as biomarkers, relating to a diverse range of measurable biological (including genomic), pathological, imaging, clinical, and physiological variables.

Diagnostic risk prediction models also exist, where the risk of already having a disease is calculated; however, the focus in this article is on predicting the risk of future outcomes. Unless the outcome prediction relates to the very near future (e.g. risk of hypocalcaemia within 48 hours after thyroidectomy [5]), single predictors usually do not provide accurate predictions at the individual-level [4]. For this reason risk prediction models usually utilise multiple predictors in combination. For example, in healthy women the probability of developing breast cancer can be estimated from the Gail model, which is a risk prediction model combining information on family history, age, age at first live birth, age at menarche, breast biopsy number, and menopause [6,7]. In women with newly diagnosed breast cancer, a well-known risk prediction model is the Nottingham Prognostic Index (NPI), [8] which gives a score that relates to the survival probability and is based on a combination of tumour grade, number of involved lymph nodes, and tumour size.

Before evaluation of its impact in daily practice [3,9,10], risk prediction model research has two main phases: model development (including internal validation using the same data or data source) and external validation (using new data from a different data source) [2,11,12]. Validation requires demonstrating that the model is accurate in the population of individuals for whom it is intended. It must ascertain the model’s ability to distinguish between patients with different outcomes (‘discrimination’) and show the agreement between predicted and observed risks in groups of individuals with similar risk predictions (‘calibration’) [1]. Importantly, validation must go beyond the set of data and individuals that were used to develop the model, because predictive performance when estimated on the development data is often optimistic, related to multiple testing with a limited sample size [1,13,14]. Validation is therefore needed in individuals not used in the development process and preferably selected from different settings (external validation) [15].

Unfortunately most publications on risk prediction models describe model development, and only a small number report external validation studies [3]. This might be a key reason why, despite many being developed, relatively few models are actually being adopted in practice. The collation and synthesis of individual participant data (IPD) from multiple studies offers a novel and natural opportunity to overcome this current lack of validation [16]. For example, models could be developed using data from a subset of studies and assessed on data from the remaining studies [17]. Variation in model accuracy across studies and its causes could also be explored. The approach would also unite researchers, increasing sample sizes and encouraging a consensus towards a single well developed and validated prognostic model, rather than a number of competing and non-validated models for the same clinical question. For example, the IMPACT (International Mission for Prognosis and Analysis of Clinical Trials) consortium developed a prediction model for mortality and unfavourable outcome in traumatic brain injury by sharing IPD from 11 studies (8509 patients), with successful external validation using IPD from another large study (6681 patients) [18].

IPD meta-analysis in this context can also go beyond using IPD from multiple studies, and more broadly consider synthesising IPD from any relevant clusters in the wider population of interest. For example, large electronic databases and registries are increasingly available that contain routinely collected patient records and risk factor measurements, which can be linked to health outcomes using, for example, Health Episode Statistics (HES) linkage. An example is the THIN database [19], which contains anonymised patient records and risk factor information from millions of patients collected from over 500 general practices in the UK [20], Such databases inevitably contain clustering of patients, for example within practices, hospitals and countries, and so an IPD meta-analysis could account for such clustering, for example by developing a model using data from a subset of the clusters (e.g. hospitals, practices), followed by external validation on the remainder.

The aim of this article is to perform a qualitative review to examine how researchers are developing and validating risk prediction models when IPD from multiple studies are sought and then combined for this purpose. The aim is to identify the current research standards and techniques; the role of IPD meta-analysis methods toward development and validation; and the common challenges and methodological problems researchers face. This allows us to generate a set of recommendations for how research in this area can be improved, and to flag those methodological techniques and issues researchers should recognise when modelling risk prediction using multiple sources of IPD.

## Methods

Our review aimed to identify and then evaluate published articles that developed and/or validated a risk prediction model using IPD from multiple studies. We now describe our review methods in detail.

### Identifying potentially relevant articles

To identify potentially relevant articles, we used an existing database of 385 IPD meta-analyses articles that was formed using a systematic review to identify all IPD meta-analyses (on any topic) published up to March 2009 [21]. The review searched Medline, Embase and the Cochrane library using a search strategy described elsewhere [22], and defined an ‘IPD meta-analysis article’ as one seeking, obtaining and then synthesising raw patient-level data across multiple studies or multiple collaborating groups. The articles in the database were published from 1991 to 2009, and it forms the largest collection of IPD meta-analyses currently available.

Note that our aim was not to review an exhaustive set of *all* risk prediction research using IPD from multiple studies, but rather to identify the main methodological methods, limitations and challenges therein. Qualitatively we felt we would achieve saturation with the existing database, and therefore we did not consider it necessary to update our review with newer articles since 2009.

### Inclusion and exclusion criteria

A relevant article was defined as one which sought and then used IPD from multiple studies to develop and/or validate a risk prediction model based on one or more predictors. There were no restrictions on the type of outcome being predicted or baseline disease/health of the patients under investigation, or the types of study (observational studies, randomised trials etc.) being utilised. We use ‘studies’ here loosely to refer to different research sources, and so it could therefore relate to different research centres or collaborating groups. However, there needed to have been a clear step for obtaining IPD from the multiple sources. We did not include articles that used an existing database already containing the multiple sources (e.g. practices). Though the analysis within such articles has similar issues, we wanted to focus on the broader picture of firstly obtaining and then analysing IPD from the multiple sources (the typical framework for an IPD meta-analysis).

Similarly ‘model’ is loosely used for any developed equation, tool, or classification approach that allowed an individual’s risk to be predicted. Articles that evaluated one or more factors for their association with outcome but not their ability to predict individual outcome risk were excluded; for example prognostic factor, risk factor and causal factor studies were excluded if they only considered factors in relation to relative risk (e.g. hazard ratios, odds ratios) and not also absolute risk (e.g. probability of death by one year).

IA screened the abstracts and titles of each of the 385 articles and classified them in regards their risk prediction model status as either ‘yes’, ‘unsure’, or ‘no’. TD then also independently classified each article as ‘yes’, ‘unsure’ and ‘no’. Finally RR checked all the ‘yes’ and ‘unsure’ articles and a random 10% of the ‘no’ articles, along with any ‘no’ article containing the words ‘prognostic model’ or ‘prognostic index’ or ‘prediction rule’ or ‘prediction model’ or ‘risk model’. Any discrepancies between the three reviewers were resolved through discussion and by obtaining the full papers. Any article deemed ‘yes’ or ‘unsure’ after this screening was then obtained and read in full by IA and TD, and a final set of relevant articles decided upon. Any discrepancies were checked by RR and a final decision was then made.

### Data extraction and in-depth evaluation of articles

Each article finally classed as a ‘yes’ was used for in-depth evaluation. A data extraction form was developed that included over 70 questions (see Additional file 1). These questions covered the rationale, conduct, analysis, reporting, and feasibility of project developing and/or validating a risk prediction model using IPD from multiple studies. A summary of the questions used is as follows:

*Background and objectives*: e.g. researchers location, year of publication, research aim, the baseline condition of patients, and the outcome to be predicted.

*Identifying IPD studies:* e.g. how they relevant studies for inclusion were identified, what types of studies were included, whether the targeted number of patients or studies was explained or justified statistically, etc.

*Obtaining IPD*: e.g. how authors asked for and obtained IPD, what proportion of IPD requested was actually obtained, whether study quality was considered, etc

*Missing data*: e.g. if there were any missing data, either at the patient-level or study-level, and if so how it was handled?

*Model development*: e.g. the statistical methods used to develop the risk prediction model, how data coming from multiple studies was handled, whether heterogeneity between studies was considered, how continuous predictors were handled, and whether the final model was fully presented.

*Model validation:* e.g. the statistical methods and criteria used for (internally or externally) validating the prediction model, how multiple studies were handled in this process, etc.

*Potential for bias:* e.g. potential impact of studies not willing/able to provide IPD on the estimates of model performance (e.g. in terms of calibration and discrimination) for the intended model’s use and target population.

*Conclusions*: e.g. the key conclusions and recommendations, and the limitations and problems discussed.

IA read each article in full and extracted information that answered these 77 questions, and then TD also independently answered these questions for each article. Any discrepancies in responses were resolved with RR.

## Results

The classification process identified 15 relevant articles each of which used IPD from multiple studies to develop and/or validate a prediction model (containing one or more predictors) for outcome risk [5,18,23-35]. They were published between 1994 and 2008, and their background and aims are summarised in Table 1. All 15 of these articles developed a model, and 11 also undertook some form of validation of their model [5,18,23,26,27,29-31,33-35]. By evaluating these 15 articles the team considered that qualitative saturation had been achieved. We now provide a qualitative summary of the key findings.

**Table 1:** Summary of the 15 articles included in the review

**First author and year of publication**	**First author location**	**Research aims**	**Baseline condition of participants**	**Main outcome(s) of interest for prediction**	**Approach to identify relevant studies**	**Number of studies providing IPD (Number requested)**
**Pagliaro (1994)**[29]	Italy	To identify predictors of short-term and sustained Alanine transaminase (ALT) normalization after interferon treatment in adult patients with hepatitis C	Adult patients with transfusion-related or community-acquired	Short term and sustained response (ALT normalization)	Collaborative group	2 (NA)
**Heffner (2000)**[26]	USA	To determine the predictive accuracy of pH for identifying patients with malignant pleural effusions who will fail pleurodesis	Patients with malignant pleural effusions	Failure of pleurodesis	Literature review	6 (12)
**Raboud (2000)**[30]	Canada	To determine the ability of intermediate plasma viral load (pVL) measurements to predict virologic outcome at 52 weeks of follow-up in clinical trials of antiretroviral therapy	Patients within a particular range of CD4 cell counts, naive to antiretroviral therapy and not been previously diagnosed with AIDS	Virologic outcome at 52 weeks of follow-up	Collaborative group	3 (NA)
**Terwee (2000)**[34]	Netherlands	To develop a prognostic tool for patients with unresectable pancreatic cancer to distinguish between low or high probabilities of survival 3 to 9 months after diagnosis.	Patients diagnosed with pancreatic cancer	Overall survival	Literature review	8 (15)
**Chau (2004)**[24]	United Kingdom	To identify baseline patient- or tumour-related prognostic factors; and to assess whether pre-treatment quality of life predicts survival in patients with locally advanced or metastatic esophago-gastric cancer.	Patients with histologically confirmed inoperable adenocarcinoma, squamous cell carcinoma, or undifferentiated carcinoma of the oesophagus, esophago-gastric junction, or stomach	Overall survival	Collaborative group	3 (NA)
**Horn (2005)**[27]	Netherlands	Investigate if transcranial doppler monitoring for micro embolic signals, directly after carotid endarterectomy (CEA) identifies patients at risk of developing ischaemic complications.	Carotid endarterectomy patients	Cerebral ischaemic complications, defined as new neurological deficits within 1st week after CEA	Literature review	7 (10)
**Nieder (2005)**[28]	Germany	Identifying the predictive value of biologically effective dose as function of the risk of myelopathy	Patients with spinal cord retreatment	Development of radiation myelopathy	Literature review	8 (8)
**Asia pacific group (2006)**[23]	Australia	To investigate the generalisability of current definitions of the metabolic syndrome in Asia-Pacific populations, and assess the prognostic value of metabolic risk factors to discriminate fatal coronary heart disease (CHD) risk	Healthy patients aged 30–75	Fatal CHD within 10 years	Collaborative group	26 (NA)
**Sylvester (2006)**[33]	Belgium	To predict a superficial bladder cancer patient’s probability of recurrence and progression at one and five years	Stage Ta, T1, and Tis bladder cancer patients who have undergone transurethral resection	Time to first recurrence (disease-free interval) and time to progression to muscle invasive disease	Collaborative group	7 (NA)
**Noordzij (2007)**[5]	USA	Early prediction of hypocalcaemia after thyroidectomy using parathyroid hormone	Patients undergoing thyroidectomy	Postoperative symptomatic hypocalcaemia	Literature review	9 (15)
**Rovers (2007)**[31]	Netherlands	To determine the predictors of a prolonged course for children with acute otitis media (AOM) to discriminate between children with and without poor outcomes	Children with AOM	A prolonged course of AOM (pain and/or fever at 3 to 7 days)	Literature review	6 (10)
**Schaich (2007)**[32]	Germany	To identify prognostic indicators in acute myeloid leukaemia (AML) to provide a new prognostic model for risk stratification of AML patients	AML patients	Overall survival and relapse-free survival	Collaborative group	8 (NA)
**Fowkes (2008)**[25]	United Kingdom	To determine if the ankle brachial index provides information on the risk of cardiovascular events and mortality independently of the Framingham risk score and can improve risk prediction	Participants of any age and sex derived from a general population	Total and cardiovascular mortality	Literature review	16 (20)
**Steyerberg (2008)**[18]	Netherlands	To develop prediction model for predicting unfavourable outcome according to the glasgow outcome scale (GOS) at 6 months after traumatic brain injury (TBI)	Patients with moderate or severe TBI (with GOS > = 12)	6-months mortality and unfavourable outcomes defined by 6 months GOS	Collaborative group	11 (NA)
**Yap (2008)**[35]	United Kingdom	To design a prognostic indicator using demographic information to select patients at risk of dying after myocardial infarction (MI)	Patients at day 45 post-MI up to 2 years	All-cause, arrhythmic and non-arrhythmic cardiac mortality within 2 years	Not stated	4 (unknown)

*NA* not applicable.

### Background and objectives

The central location of the 15 IPD projects (where the first author was located) included the USA and Australasia, but most were from Europe (11), especially The Netherlands (4) and the UK (3). Just three of the 15 articles referred to a protocol for their IPD project, and only six mentioned obtaining ethics approval.

Thirteen articles considered patients who were diseased at baseline; for example Sylvester et al. [33] estimate a superficial bladder cancer patient’s probability of recurrence and progression at one and five years. The other two articles considered patients who were healthy at baseline (Table 1); for example Fowkes et al. [25] predict the risk total and cardiovascular mortality in healthy individuals. The diseases considered at baseline included pancreatic cancer, chronic hepatitis C, bladder cancer and post-myocardial infarction among others (Table 1). The outcomes being predicted were either general (e.g. mortality) or disease-specific (e.g. development of radiation myelopathy, fatal coronary heart disease within 10 years, postoperative symptomatic hypocalcemia) (Table 1).

Thirteen of the 15 articles primarily described a multivariable risk prediction model; that is, a model which included multiple predictors. The other two articles examined the predictive accuracy of a single predictor. Noordzij et al. [5] assessed the accuracy of % change in parathyroid hormone (PTH) levels from pre- to post-thyroidectomy for predicting hypocalcemia within 48 hours, whilst Raboud et al. [30] consider the accuracy of plasma viral load (pVL) to predict virologic outcome at week 52 in patients receiving antiretroviral therapy.

### Obtaining IPD from multiple studies

Across the 15 articles, there were two competing approaches to initiating risk prediction model research using IPD from multiple studies: either perform a (systematic) literature review and seek IPD from relevant studies identified (seven articles) [5,25-28,31,34], or set-up a collaborative group of selected researchers who agree to share their IPD (seven articles) [18,23,24,29,30,32,33]. In one article it was unclear how IPD studies were identified [35].

**Figure 1:**
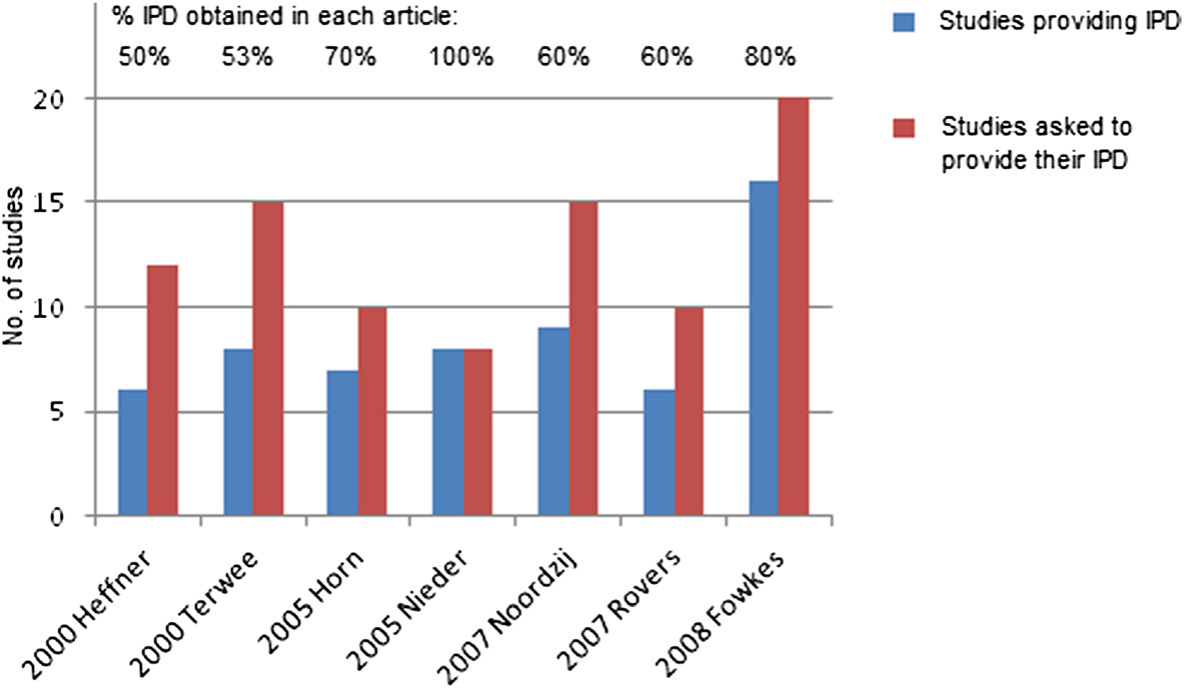
Number of studies for which IPD was requested and obtained in the seven articles using a literature review to identify relevant studies to seek IPD from.

Of the seven articles using a literature review, only three described how they contacted study authors to obtain their IPD and this included e-mail, postal mail, and telephone. Six of the seven articles did not obtain IPD for all studies desired, and only three of these six articles explained why they could not obtain all IPD desired. For example, Heffner et al. [26] stated that some IPD requested was no longer available or had not been saved in some studies. The % of IPD obtained in the seven articles ranged from 50% to 100% (Figure 1), with the median 60%.

### Details of IPD obtained

The types of studies providing IPD were stated by 12 of the 15 articles. Seven articles used IPD from only randomised controlled trials (RCTs), four used only observational studies, and the remainder (Steyerberg et al. [18]) used from both RCTs and observational studies. In the eight articles that used RCTs, five used the data from all treatment groups, two just used the placebo group, and Steyerberg et al. used all the data in their primary analysis but just the placebo group for their secondary analysis.

A summary of the sample population (i.e. mean and summary patients characteristics) were given for each IPD study separately in eight of the 15 articles (e.g. Pagliaro et al. [29]), whilst five articles gave a summary just across the overall IPD, and two articles did not give this information. Only one of the 15 articles mentioned performing an assessment of quality for studies considered, which was Rovers et al. [31], although it was not clear if this had implications for the IPD they ultimately analysed.

Fourteen of the 15 articles gave the number of patients within each of the IPD studies, and the remaining article (Rovers et al. [31]) gave just the overall number of patients across all studies. Seven of the 15 articles gave the total number of events for each predicted outcome within each of the IPD studies, (e.g. Yap et al. [35]), whilst six just gave the overall number of events across all studies and two articles did not give this information at all (e.g. Raboud et al. [30]). Only four of the 15 articles gave the number of events per candidate predictor to be considered for inclusion in the model. Two of these gave this information within each IPD study separately, and the other two provided this information just across all studies. Nine of the 15 articles reported the number of events per candidate predictor in each IPD study, whilst three others gave this in terms of the overall IPD across all studies, and the remaining three did not report this information.

### Missing data

Eight of the 15 articles mentioned having missing patient data for some variables. For example Noordzij et al. [5] state that only six of the nine studies that supplied IPD obtained preoperative PTH values, and Fowkes et al. [25] that say 3.9% of the overall values were missing in their data. Eight of the 15 articles also reported having missing whole variables in some of their IPD studies; for example, Steyerberg et al. [18] state that “*pupillary reactivity was not recorded in two trials*”.

If an article reported that missing patient data occurred for a variable, then generally the article either stated that patients with missing data were excluded from the analysis (e.g. Heffner et al. [26]), or they used a (multiple) imputation approach (e.g. Steyerberg et al. [18] and Rovers et al. [31]). Additionally three of the 15 articles entirely deleted some studies due to absence of a particular predictor or even outcome of interest. For example, Terwee et al. [34] excluded three of their eight studies, stating: “*one study was excluded because we could not distinguish between patients with tumours of the pancreatic head and tumours of the pancreatic body or tail, and two others were excluded because information on metastases was lacking or incomplete*”.

### Model development

We now summarise the statistical analysis methods used for model development. Our main focus here is on how IPD from multiple studies were handled.

#### ***Analysis method***

The number of patients and events used from each study *separately* towards the prediction model development was given for 10 of the 15 articles, (e.g. Yap et al. [35]), with the remainder focusing on the numbers overall.

The two articles that considered a single predictor examined predictive performance by calculating ROC curves from the data. In the 13 articles developing a multivariate model, six used Cox regression (e.g. Terwee et al. [34]), five used logistic regression (e.g. Heffner et al. [26]), and Patel et al. [23] used both Cox and logistic regression. Nieder et al. [28] did not state the analysis method used.

All 15 articles used the IPD from multiple studies to develop their models (i.e. none used just one study for development). Ten of the 15 articles pooled all the IPD available into one big dataset and analysed it ignoring clustering of patients within studies or collaborative groups (this is often referred to as a ‘one-stage meta-analysis ignoring clustering’ [36]), mostly without explaining why clustering was ignored. A notable exception is Terwee et al. [34], who justify their approach by examining whether stratification by study was necessary, stating: *“The homogeneity assumption was checked by including treatment as a dummy-coded variable into both models (stratification per study). Initially we found a significant survival benefit for patients treated by surgical bypass procedures compared with endoscopic stents. However, this effect disappeared after adjustment for Karnofsky’s index (a measure of functional status) in the study in which this variable was available, which legitimises pooling”.*

Three of the remaining five articles developed their model via a ‘one-step meta-analysis analysis accounting for clustering’, where the IPD from all studies/collaborative groups are analysed together but with clustering by study/group accounted for (e.g. using a dummy variable for study, such as Steyerberg et al. [18]). In another article, Fowkes et al. [25] developed their model using a two-step meta-analysis approach, where the IPD are first analysed separately in each study, and then their model estimates are pooled together in second-step. Specifically, they estimate a Kaplan Meier curve for each study, and then do a random effects meta-analysis of the study survival percentages estimated at different time-points (to give an average % survival predicted across studies). In the remaining article, Schaich et al. [32] use a hierarchical cluster approach, but it is not clear whether this cluster approach specifically included clustering by study.

#### ***Heterogeneity of predictor effects***

Twelve articles did not consider between-study heterogeneity in the predictive effect of their included predictors. Of the three articles that did consider such heterogeneity, Steyerberg et al. [18] state: “*Similarly, study-specific effects were assessed with interaction terms between study and each predictor. Interaction terms between predictors were examined with likelihood ratio tests, but none was of sufficient relevance to extend the models beyond the main effects for each predictor*”. Rovers et al. [31] assessed heterogeneity through the I^2^ statistic and state: “*To determine whether pooling was justified, heterogeneity between studies was assessed with the I*^*2*^*statistic. Because the I*^*2*^*value was 25%, pooling was performed”.* Fowkes et al. [25] tested for heterogeneity (using Chi-square test and I^2^), for one of their predictors; they then applied random effects in their analysis which, although not explicitly stated, is likely due to heterogeneity being detected.

#### ***Handling of continuous predictors***

Continuous predictors were analysed on a continuous scale in four of the 15 articles. For example, Steyerberg et al. [18] state: “*For the continuous predictor’s age, glucose, and Hb, a linear relationship with outcome was found to be a good approximation after assessment of non-linearity using restricted cubic splines*”. The remaining 11 articles either categorized or dichotomized the continuous predictors of interest. For example, Heffner et al. [26] state that “*continuous variables were entered as dichotomous indicator variables with test thresholds determined by ROC analysis*”, and Chau et al. [24] state: “*Laboratory variables were initially coded as continuous variables and subsequently dichotomised with the cut-off points chosen at the median value of each variable*”.

#### ***Need for standardisation***

Three of the 15 articles mentioned the need to standardise the coding of predictors and outcome definitions across studies. For example, Terwee et al. [34] state “*To standardise definitions among the different studies, the presence of metastases and of pain and weight loss at diagnosis were classified as ‘present’ or ‘absent’*”, whilst Noordzij et al. [5] handle different methods of measuring PTH values by analysing % change in PTH from baseline, rather than analysing PTH on its original scale. In order to use and compare candidate predictors factors measured on different continuous scales, Steyerberg et al. [18] standardised the reporting of odds ratios so that they corresponded to a change from the 25th percentile to the 75th percentile of the predictor distribution.

#### ***Strategy for inclusion of predictors***

Of those 13 articles developing a multivariable model with multiple candidate predictors for inclusion, the most common approach was to use p-values to decide which factors were included. For example Heffner et al. [26] state: “*Variables that were found by univariate analysis with p < 0.10 were entered in the model. Variables were removed from the model if their p values were > 0.05. This process identified 4 clinical variables. The four clinical variables were entered into the logistic regression, and only pH was identified as an independent predictor*”. Four of the articles used a selection procedure such as forwards, backwards or stepwise selection.

#### ***Reporting of developed model***

Of those 13 articles developing a multivariable model, the developed model was poorly reported on its original scale. For example, of the six articles that used a Cox model, only Terwee et al. [34] gave the beta (log hazard ratio) estimates for each included predictor, with others mainly reporting hazard ratios for each variable. None of those six articles that used logistic regression reported the alpha term (baseline risk) for their full model, and only two reported the beta (log odds ratio) estimates for each predictor, with the remaining four articles presenting odds ratios. However, Steyerberg et al. [18] do present a simplified version of their logistic regression model (based on a simple score chart), in which they do present the alpha and beta terms.

### ***Model validation***

We now consider if and how articles validated their developed model. We refer to ‘internal validation’ when the same data are used to validate the model as to develop it, and ‘external validation’ when the data are used to validate the model are different than the data used for development. We also refer to ‘internal-external cross-validation’ [16,17], which is a multiple validation approach that accounts for multiple studies by rotating which are used toward model development and validation. Briefly, this technique excludes one of the IPD studies from the available set, and the remainder are used to develop the prediction model; the excluded study is then used to validate the model externally. This process is repeated for each study omitted in turn, and this allows the consistency of the developed model and its performance to be examined on multiple occasions. If the model performs consistently well in all external datasets, then all IPD studies can be used for model development. If, however, it performs poorly in some external datasets, this may flag up heterogeneous studies (populations) for which the model does not generalise to and thus warrant exclusion.

#### ***Internal, external, or internal-external***

Four articles did not use any validation approach. Nine of the other 11 articles performed internal validation, using the same IPD as that used to develop the model. For example, Noordzij et al. [5] examined the accuracy of PTH by calculating the ROC curve in all of the IPD available. However, a few of these nine also recognised that model accuracy may be over optimistic in the development data, and so tried to correct for this. For example, Paglioro et al. [29] took a random test set of 100 patients from their development data of 261 patients; they show that the area under the ROC curve was slightly lower in the test data than than the full original data (e.g. their model 1: AUC =0.728 (n = 261) vs. AUC = 0.659 (n = 100)). Sylvester et al. [33] performed bootstrap resampling of all the IPD available and then examined model performance in these samples, leading to a bias-corrected C-statistic.

Steyerberg et al. [18] used both external validation and internal-external cross-validation. For external validation they use IPD from a trial different than that used to develop the model, but note problems with missing variables: “*We aimed to validate all models externally using data from selected patients in the CRASH trial. However, lab values were not recorded in this trial, nor were hypoxia, hypotension, and EDH. We therefore validated the core model, and a variant of the extended model, in which only the Marshall CT classification and presence of tSAH were added to the core model (i.e., the core + CT model)”.* Interestingly, their developed model was stratified by study and so had multiple intercepts to choose from. However, in their validation they appear to choose one intercept related to a single trial as *“it represented typical proportions of mortality (278/1,118, 25%) and unfavourable outcome (456/1,118, 41%)”.*

When performing internal-external validation, Steyerberg et al. [18] state: “*AUC was calculated in a cross-validation procedure, where each study was omitted in turn. Results were pooled over the ten imputed datasets for eight studies with sufficient numbers for reliable validation (n > 500)*”. Yap et al. [35] also used internal-external cross-validation, stating: “*The use of several different studies provided an opportunity to validate the general method using data drawn from a different patient population using an internal–external cross-validation system proposed by Royston et al. that leave-one-out cross-validation on a cohort basis. We therefore, sequentially designated one of the trials as a test study. The remaining studies acted as a training data set, which was used to generate the risk scores*”.

#### ***Reporting of validation criteria***

Validation criteria focused on discrimination, calibration, and goodness of fit. Of the 11 articles that examined validation, 10 gave discrimination statistics such as sensitivity, specificity, AUC and C-statistic. Three articles reported calibration statistics such as the Hosmer-Lemeshow goodness-of-fit test. Eight provided figures to show model discrimination, accuracy, or calibration. The most common figure was an ROC curve, but Steyerberg et al. [18] present calibration figures depicting predicted and actual outcome probabilities, and Yap et al. [35] presented survival curves for each split-validation sample, to illustrate the consistency of the model in identifying risk categories.

### Dealing with those studies not willing/able to provide their IPD

In six of the seven literature review articles (Figure 1), IPD had not been obtained for all desired studies, but only three of these discussed whether this was a limitation of their project. For example, Horn et al. [27] note: “*We may not have identified all centres, which monitor MES after carotid surgery: it is likely that small series of patients remain unpublished and have therefore escaped our attention*”. None of the six articles gave the number of patients and events in the missing IPD studies, and only one article discussed the qualitative or quantitative differences between those studies providing IPD and those studies not able to.

### Conclusions and limitations

The developed risk prediction models had large potential for use in clinical practice, according to the discussion of the 15 articles, highlights the value of collecting IPD from multiple studies. For example, Noordzij et al. [5] state “*PTH assay, when checked 1 to 6 hours after thyroidectomy, has excellent accuracy in determining which patients will become symptomatically hypocalcemic*”, whilst Yap et al. [35] state “*our study suggests that in post-MI patients, pre-selected using LVEF or frequent ventricular premature beats, the additional use of a simple prognostic indicator based on demographic and baseline information was able to segregate patients that were at high risk of dying, for 3 different modes of mortality*”.

However, numerous limitations and problems of the IPD projects were also noted in the articles’ discussion section, especially dealing with missing data and between-study heterogeneity in definitions, time-periods, treatments used, study quality, and different methods of measurement. The key methodological problems are summarised in Table 2.

**Table 2:** **Methodological challenges when developing and validating a risk prediction model using IPD from multiple studies as identified from those 15 articles in our review (written below in a framework similar to recommendations by Abo-Zaid et al.**[37]**for prognostic factors)**

**Methodological issue**	**Challenge**
**Identifying relevant studies**	• Unavailability of IPD in some studies
**Issues within studies**	• How to assess quality of studies available
	• Inability of IPD to overcome deficiencies of original studies, such as missing participant data or of being low methodological quality.
**Heterogeneity across studies**	• Dealing with different definitions of disease or outcome
	• Dealing with different (or out-dated) treatment strategies, especially when a mixture of older and newer studies are combined
**Statistical issues for meta-analysis**	• Dealing with a mixture of IPD from retrospective and prospective studies
	• Missing data, including: missing predictor values and missing outcome data for some participants within a study, and completely unavailable predictors in some studies
	• Difficulty in using a continuous scale for continuous factors in meta-analysis when some IPD studies give values on a continuous scale and others do not
	• Dealing with IPD from trials where both control and treatment groups are available
**Assessment of potential biases**	• How to assess the impact of excluded studies who did not provide IPD
**Model development**	• Accounting for clustering of patients within different IPD studies
	• Allowing for heterogeneity in baseline risk (intercept term) across studies
	• Allowing for heterogeneity in predictor effects across studies
**Model validation**	• Lack of external validation if all studies used for model development
	• Sample size required to implement the internal-external approach (i.e. sample size of studies to be excluded, and also the total number of IPD studies needed)

## Discussion

Risk prediction models have the potential to inform strategies for disease prevention, early diagnosis, patient counselling and therapeutic care [1]. As for all clinical practice, their use should be evidence-based. In particular, there must be consistent evidence that a model is reliable and applicable to the intended populations of individuals [3]. This ideally requires the model to be successfully validated in multiple datasets external to the model development phase. This can often take many years to achieve. However, with increasing access to the IPD from existing studies or large databases, there is a growing opportunity to both develop and validate risk prediction models simultaneously, within an IPD meta-analysis framework [16].

In this article we have reviewed 15 articles that each used IPD from multiple studies to develop and potentially validate a risk prediction model for the development of future outcomes. This has allowed us to identify good practice, useful statistical methods, methodological challenges (Table 2), and some limitations in current reporting and methodology to be addressed (Table 3). We recognise that our review also has limitations. Firstly, it only covers articles published up to 2009, which was a restriction on the database we used; however we feel that our findings are unlikely to be different if the review were updated, and that qualitative saturation of issues and concepts had been achieved with our sample. Secondly, we focused on articles that utilise IPD obtained from multiple studies (or sources), and did not consider articles which used a single database containing clustering (e.g. by practice); such articles raise similar issues for the analysis, but we wanted to focus on the typical IPD meta-analysis scenario where IPD studies are obtained and then synthesised. Thirdly, by evaluating published articles we recognise our findings are clearly dependent on the reporting standards within the articles; thus any apparent research deficiencies or methodological gaps may just reflect poor reporting standards. Nevertheless, we believe our review and its findings will help inform those who wish to develop or validate a model using IPD multiple studies in the future. In particular, our work allows us to provide some key recommendations for improving the design, conduct and reporting of future research this field (Table 3), which echo those for IPD meta-analysis of prognostic factor [37] and modelling [16] studies, and concur with previous [38,39] and ongoing [40] work toward improved reporting of risk prediction model articles in general. For brevity we do not discuss all these in detail now, but rather focus on the two most important recommendations in detail.

**Table 3:** Recommendations for improved research when developing and validating risk prediction models from multiple studies

**Area for improvement**	**Recommendations**
**Rationale and initiation**	• Produce a protocol for the project, detailing rationale, conduct and statistical analysis and reference this
**Obtaining IPD**	• Report how the primary study authors were approached for their IPD
	• Report strategy used to identify relevant studies, e.g. literature review/collaborative group
	• If literature review performed, then report search strategy, including keywords and databases used
	• Provide a flowchart showing the search strategy, classification of identified articles, and retrieval of IPD from relevant studies
	• Report any prior sample size considerations used, such as the number of IPD studies deemed necessary and the number of patients and events required. If no sample size requirements were considered, report this also
**Details of IPD**	• Report the number of patients and events for each study used in model development and/or validation
	• Report the missing data for each study (e.g. whether predictors were missing entirely, or how many patients had predictor values missing), and whether some patients or studies were entirely excluded for this reason
	• Detail the reasons why IPD was unavailable in some desired studies (if applicable), and report the number of patients and events from these studies
	• If any studies were excluded after IPD was obtained, provide the number of studies excluded and explain why they were removed (e.g. missing predictors, different outcome definition, different methods of measurement)
	• Compare and report the quality of studies for which IPD was obtained
**Statistical methods for model development**	• Account for clustering of patients within studies, for example by allowing for a separate intercept per study
	• Report the selection criteria and procedure used to decide which predictors are included in the final model
	• Assess and report any between study heterogeneity in the effects of included predictors
	• If large heterogeneity does exist in particular predictors, then try to reduce it by including more predictors or simply focus on including homogenous or weakly heterogeneous factors
	• Where possible model continuous predictors on their continuous scale, unless it is important to categorise with good clinical or statistical reason
	• Report the final developed model in original format with alpha (baseline risk) and beta estimates, so that others can ascertain how apply the model in practice
	• Detail how missing patient-level data and missing study-level factors were dealt with in the analysis
**Model validation and implementation**	• Validate the model that has been developed using internal-external cross-validation; we tentatively suggest at least 4 studies are required for this approach however.
	• Explain the choice of intercept (baseline hazard) to be used when implementing the model in the excluded study
	• Report validation statistics for each study excluded in the internal-external cross validation method
	• Report clearly whether there is evidence the model performs consistently well during the internal-external validation
	• If it performs consistently well, clearly report the final overall prediction model to be used in practice, and emphasise again how the intercept should be chosen upon application
	- If it does not perform consistently well, clearly flag those populations for which the model cannot be applied and draw attention to the model’s lack of generalisability
**Impact of missing IPD studies**	• If possible, compare the populations of those studies not providing IPD to those studies providing IPD, to be able to understand whether the developed model may need further generalisation in such populations in the future

### Recommendation 1: allow for different baseline risks in each of the IPD studies

In our review, 10 of the 15 articles did not account for clustering of patients within different IPD studies and therefore their developed prediction model did not allow for any study differences in baseline risk. Although such models can still perform adequately on average (that is, across all studies combined), when applied in practice to particular populations the model performance may deteriorate considerably if the population’s baseline risk is very different from the average estimated during model development. In other words, the developed model may require re-calibration in particular populations. Statistically it is also know than omission of an important predictor (i.e. study) can lead to biased effect estimates and reduced power [36]. To address this, Debray et al. [16] recommend that the prediction model should be developed with a separate intercept (baseline risk) per study, and then the model’s performance can be examined using internal-external validation alongside a strategy for choosing the intercept upon application to the excluded study. Such strategies may include using external knowledge of the intercept in the excluded study population; using the intercept as estimated from the IPD from the excluded study; or taking the intercept estimated from a study used in the model development that contains a similar population to the excluded study. The latter strategy is recommended within Steyerberg et al. [18], where they propose others apply their model using the intercept for one particular trial in their analysis, as this trial is reflective of the population intended.

Where the intercept can be well-matched to the excluded study, Debray et al. [16] show that their framework allows an IPD meta-analysis to produce a single, integrated prediction model from that can be implemented in practice and has improved model performance and generalisability. This echoes other recommendations to account for clustering in an IPD meta-analysis [36]. For survival data, this means that the baseline hazard should be modelled during model development and so researchers should move away from using Cox regression (a common approach in the articles in our review, but one which does not estimate the baseline hazard) and rather use other approaches such as flexible parametric methods, like the Royston-Parmar model that estimates the baseline hazard using restricted cubic splines [41,42].

A similar recommendation is for researchers to examine between-study heterogeneity in predictor effects. This is currently rarely done. Debray et al. [16] show that a model’s performance is likely to be more consistent if there is little or no heterogeneity in the effect of the predictors included. Researchers should examine heterogeneity and prioritise inclusion of homogeneous and only weakly heterogeneous predictors, or attempt to include interaction terms or additional predictors that reduce heterogeneity in others. The choice of predictors and their specification in the model (e.g. with transformation, and/or with linear or non-linear trends) is a complex issue, and statistical software procedures to integrate such decisions in the context of an IPD meta-analysis would be very helpful.

### Recommendation 2: implement a framework that uses internal-external cross-validation

A major finding from our review is that, despite the availability of multiple studies, most researchers develop their model by using the IPD from all available studies, and so then perform an internal validation (on the same set of data) rather than an external validation (on different data). Only two of the 15 articles used a form of external validation, and so most models require further validation in order to investigate their true performance. One plausible reason why researchers choose not to use IPD for external validation is that they want to maximise the data available for model development; this is understandable, especially when faced with a large set of candidate predictors and possible non-linear relationships. Furthermore, even if researchers do decide to hold-back some IPD for external validation, it is not easy to decide how much IPD (and how many studies) should be removed.

For these reasons, the internal-external cross-validation approach is highly appealing [16,17], yet seemingly under-utilised. It wasused by Steyerberg et al. [18] and Yap et al. [35] in the articles we reviewed up to 2009. We also performed a citation search of the Royston et al. [17] article that proposed the method. By the end of 2012 (based on abstracts of citations identified) there were still only nine citing articles (including the aforementioned Yap and Steyerberg) that developed a prediction model.

The internal-external cross-validation approach involves removing just one study from the development phase of the model, fitting the model on the remaining IPD, and then testing performance in the excluded study. This framework is then repeated by rotating the omitted study and assessing the validation in all the possible scenarios. Model estimates are therefore always based on the majority of IPD, and its model fit and predictive ability can be appraised across all the studies simultaneously. Where performance is consistently adequate across all combinations of the omitted study, a final step can be to utilise the IPD from all studies to produce the final specification of the model. In situations where model fit appears inadequate in some excluded studies, then this identifies a lack of generalisability and highlights populations (studies) for which the model is not currently suitable for. It also signals the need to improve the current model specification, for example by including additional predictors with homogenous effects across studies.

Royston et al*.*[17] originally proposed the internal-external validation approach for survival data, within a framework to construct and validate a prognostic survival model from an IPD meta-analysis [12]. This framework allowed them to evaluate whether derived models have good prognostic separation in independent studies and whether the baseline survival distribution is heterogeneous across studies. Afterwards, a single final model was derived from all available IPD using flexible parametric proportional hazards (PH) modelling techniques. This article has only been cited 26 times between 2004-2012, again showing the general lack of uptake of this method. We hope this will change as researchers become more aware of its usefulness. Debray et al. [16] recently extended the methodological framework to binary outcomes where logistic regression models are used to develop the risk prediction model.

### How many IPD studies are needed and should all available IPD be used?

We recognise that implementing these two recommendations may be difficult given only a few IPD studies and/or when the number of patients per study is small. In particular, there is a need for methodological research on the necessary sample size to implement the internal-external approach, in terms of both the total number of IPD studies needed (Debray et al. [16] tentatively suggest at least four or five) and the sample size (number of patients and events) within those studies that are excluded from model development. With small sample sizes, internal-external validation may not be plausible; for example, Nieder et al. [28] only obtained IPD for 40 patients in total across eight case series reports. However, such small sample sizes are perhaps less likely to be an issue when using data from trials, prospectively planned cohort studies, or large electronic databases.

Given a set of available IPD studies, researchers should begin by identifying those that are relevant and most reliable for the clinical question at hand. It may be entirely sensible to exclude some available IPD for a variety of reasons framework [16]. For example, researchers should evaluate the outcome definitions used in each study, and exclude studies that are not consistent (and cannot be made consistent) with others. They should also check whether important predictors are recorded in each study, and evaluate the amount of missing data for available predictors; though multiple imputation can limit these issues, studies with multiple missing predictors or large proportions of missing values might be best excluded for robustness (unless imputation assumptions can be justified). Studies may also be removed if they have crucial differences in such as: the method of measuring an important predictor; the treatment and healthcare patients received during follow-up; and the start-point (baseline) entry of patients to the study (e.g. one year after treatment, rather than at the time of diagnosis). If ignored, such differences might limit clinical interpretability of any model produced and reduce its performance. The number of studies excluded, and the decisions that lead to this, should always be transparently reported upon publication (Table 3).

A further issue arises when IPD are not available for all desired studies. This raises the threat of availability bias, where studies not providing their IPD are potentially different to those that do provide IPD [43]. For a traditional meta-analysis of treatment effects, this can cause bias in the summary meta-analysis result [43]. In the context of risk prediction models, it might cause the developed model to be unreliable in those populations (studies) which did not provide their IPD, and therefore reduce its usefulness in practice until further validation studies can be undertaken in these populations.

Finally, an issue rarely considered is whether ethical approval is required to collate IPD from multiple studies. Only six of the 15 articles mentioned that had ethical approval. One might argue that ethical approval is not required when IPD is being used in accordance with the original objectives of the studies involved (that is, to understand and improve the prognosis of patients). We hope that most ethics committees will support this view, but it should at least be checked that the studies providing IPD actually had ethics approval themselves.

## Conclusions

It is paramount to consider statistical and methodological issues when planning to develop and/or validate a risk prediction model from multiple datasets, in order to avoid poorly generalisable and poorly performing models. Our review highlights that the IPD meta-analysis approach is highly appealing, as it allows the use of internal-external cross validation to develop a model and simultaneously evaluate its performance across multiple populations. However, researchers are faced with numerous challenges when the IPD is collated, in particular missing data and heterogeneity in study quality and methods of measurement. Perhaps an ideal way forward is a prospective IPD meta-analysis, where researchers agree at their study onset to use set quality standards and record particular variables in a common way so that, upon their own study completion, they can supply their IPD to those developing/validating a risk prediction model. Heterogeneity can then be limited by researchers agreeing, before data collection, to standardize predictor definitions, measurement methods, and outcome recoding.

Our review has only considered articles that developed or validated a prediction model. For risk prediction models to become more common in practice, research also needs to show they have a positive impact on health outcomes. Such impact studies are currently rare [3], but they should follow any IPD meta-analysis that develops and validates an accurate risk prediction model.

## Competing interests

The authors declare that they have no competing interests.

## Authors’ contributions

RR and KM conceived the study. IA developed the questions for the data extraction form, which were then refined and extended by all authors. IA and TD independently extracted information from the articles to answer each question. IA collated responses, and resolved any disagreements with TD and RR. IA wrote the results up in full for his thesis, including tables and figures. RR converted this chapter into the form presented in this article, and this was edited and added to by KM, TD and IA. All authors contributed to revisions of the article in response to reviewers’ comments. All authors read and approved the final manuscript.

## Pre-publication history

The pre-publication history for this paper can be accessed here:

http://www.biomedcentral.com/1471-2288/14/3/prepub

## Supplementary Material

Additional file 1: Full list of questions used to evaluate the 15 articles. Click here for file
