# Supplementary material for: Developing and validating risk prediction models in an individual participant data meta-analysis
Source: BMC Med Res Methodol. 2014 Jan 8;14:3. doi: 10.1186/1471-2288-14-3 (PMC3890557; doi:10.1186/1471-2288-14-3)
Supplement: Additional file 1: — Full list of questions used to evaluate the 15 articles. [file 1471-2288-14-3-S1.docx]

**Appendix**: **Full list of questions used to evaluate the 15 articles**

**1) Background information**

- What country is the corresponding author located in? (i.e. what is the central location for the IPD project?)
- Is there a reference to a *protocol* for the IPD project, and, if so, were details given as to where it can be found?
- How the project was funded, and was *ethics approval* granted for the IPD project? If not,
  were reasons given as to why ethics approval was not necessary and, if so, what were the reasons?
- Number of studies/datasets included?
- What were the types of different studies included (e.g. studies, databases etc)?
- Number of authors/researchers?

**2) Research objectives**

- What was the key research aims of the paper in relation to prognosis/risk prediction?
- At baseline what was the condition of the patients being assessed?
  (E.g. what disease did they have, or what operation had they just had, or were they healthy etc, Note: If diagnostic prediction model; than suspicion of the disease is starting point).
- What outcomes or diseases were of interest for prediction?

**3) Identifying studies and their IPD**

- What was the process used to identify relevant studies for the IPD project?
  (E.g. literature review or collaborative group).

1. If a literature review, then what search strategy was used (e.g. search of Medline, Embase) and was a list of keywords given? Also was a flow chart shown, giving the flow of studies into and out of the project?
2. If collaborative group, how were studies or databases chosen to be included in the collaborative group?
    (e.g. friends in the field, existing database, etc)

- What are the types of studies for inclusion?, e.g. RCTs, just placebo arms from RCTs, cohort study, hospital databases, insurance company registries etc
- Was the total sample size or total number of studies required for the model development and/or validation justified? E.g. sample size calculation, or did they ask for IPD from all studies available?
- Also info on number and types of outcomes, as these are related to power considerations together with the number of candidate predictors?
- And most importantly perhaps: number of pre-planned subgroup analyses (as this is the main idea for IPDs in at least therapeutic studies)

**4) Asking for and obtaining IPD**

- How were authors of relevant studies approached for IPD (e.g. e-mail, letter, phone etc.)?
- How many studies (or collaborating groups) were ultimately approached for IPD, and what proportion of these studies/groups actually provided IPD?
- (If appropriate) what were the reasons given as to why some studies refused to provide IPD?
- How many studies ultimately provided IPD?

1. What types of studies were they (e.g. RCT, cohort, etc)?
2. Was the number of patients within each of the IPD studies given? If not, was the number of patients across all studies given?
3. Was the total number of events given for each predicted outcome, within each of the IPD studies? If not, was the total number of events given across all studies?
4. Was the number of events per candidate variable (predictor) given in each study? If not, was the number of events per variable given across all studies?
5. Was the number of candidate predictors in total and per study given?
6. Was a summary of the sample population given for each study separately (e.g. mean age, proportion male, treatments in use etc); if not, was it given for the whole IPD combined?
7. Did they synchronise prediction and outcome definitions and measurements?
8. Did they have to do that?
9. What attempts were made?
10. Did they use proxies if not exactly same measurement method or definition?
11. Did they delete studies due to absence or completely different predictor or outcome?

- Did the inclusion / exclusion criteria for an IPD study include an assessment of study quality?

1. If yes, what quality criteria were used to decide inclusion or exclusion (or ‘low’ quality and ‘high’ quality)?
2. Were IPD still sought from low quality studies?

**5) Missing data**

- At individual-level: Were details of any missing individual-level data within the available IPD given for each study, and, if so, what were they? (e.g. for some patients their age was unknown) (Note: Check tables to help with this)
- At study-level: Did all studies have all candidate predictors or outcomes of interest, or was there any missing data across studies? (e.g. for some studies, age was not recorded at all)
- If either of these were detailed, how was missing data handled in the analysis?

**6) Model development: Statistical analysis methods**

- Are any articles referred to for methodology or any statistical methods cited? Especially if it relates to using data from multiple studies. (Note: for my own reference mainly)
- Was a statistical analysis plan for model development given or mentioned in the Methods section?
- Was the number of patients and events used from each study toward the prediction model development given?
- Were the prediction models developed using the IPD from multiple studies? If so, how was the data synthesised:

1. ‘one-step ignoring clustering’: lumping all the data together into one big dataset, and ignoring clustering by study or collaborative group; or
2. ‘a one-step analysis accounting for clustering’, where the data from all studies/collaborative groups are analysed together but with clustering by study/group accounted for (e.g. using a dummy variable for study); or
3. A two-step approach, where the data are first analysed separately in each study, and then their model estimates are pooled together in second-step.
4. Developed using a part of the dataset, and then validated using the remaining data

- What types of statistical models were used?

1. In the two-step approach, details are needed here of how individual studies were analysed, and then how the model estimates were pooled using meta-analysis.
2. In the one-step approach, again details are need here of the one-step model itself (Cox regression, logistic regression) and the meta-analysis assumptions therein (e.g. fixed or random-effects on the predictor effects)

- Was between-study heterogeneity in the predictor/outcomes considered within the prediction model? If so how (e.g. using random-effects in the analysis; assessing heterogeneity using I-squared)
- How were continuous predictors in the prediction model analysed, on a continuous scale or categorized?
  If categorized

1. Were reasons given as to why this was done?
2. How many cut-points were used, and how were they chosen?

If on a continuous scale

1. Were non-linear trends assessed and, if so, how were they modelled? (e.g. splines, fractional polynomials)
2. Was the continuous factor analysed on its original scale, or was it on a transformed scale and why?

- If multivariable models were fitted (i.e. prediction models that included multiple variables)

1. What criteria were used to decide inclusion of a predictor in the model? (e.g. statistical criteria, such as p< 0.1, or clinical criteria such as a hazard ratio > 2 or inclusion of ‘smoking’ variable regardless
2. What selection procedure was used? (e.g. forward, backward, stepwise)
   - Was the final model given in full (i.e. with parameter estimates and standard error or CI for each)? If not, what was given?
   - List all the problems that are stated or evident that limited the statistical analysis (E.g. different method of measurements, different predictors available in each study etc) and how did the authors attempt to overcome these problems?

**7) Model Validation**

- Internal validation (using the same data used to generate the model)
- Was this done?
- If so, how?
- How good was the performance of the model after the validation?
- External validation (using different data than that used to generate the model)e.g. data from other centres not used to develop the model, or half of the data from each centre that was not used to develop and left to validate instead, etc.)
- Was this done?
- If so, how?
- How good was the performance of the model after the validation?
- What discrimination, calibration, reclassification or other statistics (e.g. goodness of fit or R2) were used?
- Were any figures given to show model discrimination / accuracy / calibration?
- If so, what?
- Were the results presented with their CI’s or S.E’s?
- Was the number of studies used in the validation stated?
- Was the number of patients and events used from each study toward the prediction model validation given?

**8) Dealing with those studies not willing / able to provide their IPD**

- (If appropriate) Was there an assessment or discussion of whether the available IPD studies were a biased set of all studies in the field?
- (If appropriate) for studies not providing IPD, were details given as to the number of patients and events in these studies
- (if appropriate) were there any other details provided on the qualitative or quantitative differences between those studies providing IPD and those studies not able to provide IPD? If so, what were these differences?

**9) Conclusions and Discussion**

- What were the main clinical conclusions of the IPD project in the Discussion, in relation to the use or implementation of the model?
- What limitations and problems of the IPD project were noted in the Discussion?
- Were totally different conclusions made given the results?
